# Rationale and design of the randomised clinical trial comparing early medication change (EMC) strategy with treatment as usual (TAU) in patients with Major Depressive Disorder - the EMC trial

**DOI:** 10.1186/1745-6215-11-21

**Published:** 2010-02-26

**Authors:** André Tadić, Stanislav Gorbulev, Norbert Dahmen, Christoph Hiemke, Dieter F Braus, Joachim Röschke, Dietrich van Calker, Daniel Wachtlin, Kai Kronfeld, Thorsten Gorbauch, Monika Seibert-Grafe, Klaus Lieb

**Affiliations:** 1 Department of Psychiatry and Psychotherapy, University Medical Centre Mainz, Germany; 2 Interdisciplinary Centre for Clinical Trials (IZKS), University Medical Centre Mainz, Germany; 3 Clinic for Psychiatry and Psychotherapy, Katzenelnbogen, Germany; 4 Clinic for Psychiatry and Psychotherapy, Dr. Horst-Schmidt-Kliniken, Wiesbaden, Germany; 5 St Valentinushaus, Clinic for Psychiatry and Psychotherapy, Bad Soden, Germany; 6 St Valentinushaus, Clinic for Psychiatry and Psychotherapy, Kiedrich, Germany; 7 Department of Psychiatry and Psychotherapy, University Medical Centre Freiburg, Germany

## Abstract

**Background:**

In Major Depressive Disorder (MDD), the traditional belief of a delayed onset of antidepressants' effects has lead to the concept of current guidelines that treatment durations should be between 3-8 weeks before medication change in case of insufficient outcome. Post hoc analyses of clinical trials, however, have shown that improvement usually occurs within the first 10-14 days of treatment and that such early improvement (Hamilton Depression Rating Scale [HAMD] decrease ≥20%) has a substantial predictive value for final treatment outcome. Even more important, non-improvement (HAMD decrease <20%) after 14 days of treatment was found to be highly predictive for a poor final treatment outcome.

**Methods/Design:**

The EMC trial is a phase IV, multi-centre, multi-step, randomized, observer-blinded, actively controlled parallel-group clinical trial to investigate for the first time prospectively, whether non-improvers after 14 days of antidepressant treatment with an early medication change (EMC) are more likely to attain remission (HAMD-17 ≤7) on treatment day 56 compared to patients treated according to current guideline recommendation (treatment as usual; TAU). In level 1 of the EMC trial, non-improvers after 14 days of antidepressant treatment will be randomised to an EMC strategy or TAU. The EMC strategy for this study schedules a first medication change on day 15; in case of non-improvement between days 15-28, a second medication change will be performed. TAU schedules the first medication change after 28 days in case of non-response (HAMD-17 decrease <50%). Both interventions will last 42 days. In levels 2 and 3, EMC strategies will be compared with TAU strategies in improvers on day 14, who experience a stagnation of improvement during the course of treatment. The trial is supported by the German Federal Ministry of Education and Research (BMBF) and will be conducted in cooperation with the BMBF funded Interdisciplinary Centre Clinical Trials (IZKS) at the University Medical Centre Mainz and at six clinical trial sites in Germany.

**Discussion:**

If the EMC strategies lead to significantly more remitters, changes of clinical practice, guidelines for the treatment of MDD as well as research settings can be expected.

**Trial Registration:**

**Clincaltrials.gov Identifier**: NCT00974155; **EudraCT**: 2008-008280-96.

## Background

*Major depressive disorder (MDD) *is a psychiatric illness in which mood, thoughts and behavioural patterns are impaired for long periods. The illness distresses the person and impairs his or her social functioning and quality of life. MDD is characterized by marked sadness or a loss of interest or pleasure in daily activities, and is accompanied by weight change, sleep disturbance, fatigue, difficulty concentrating, physical impairment and a high suicide rate [1].

### Prevalence of MDD

MDD is a highly prevalent, often chronic or episodic lifelong disorder. The recent large, community-based European Study of the Epidemiology of Mental Disorders (ESEMeD) found major depression being the most prevalent psychiatric disorder with lifetime and 12-months prevalence rates as high as 12.8 and 3.9%, respectively, in the total population. In females the prevalence rates were almost twice as high as compared to men (lifetime: 16.5% vs. 8.9%, resp.; 12-months: 5.0% vs. 2.9%, resp.) [2]. In the United States (US) the recent National Comorbidity Survey Replication (NCS-R) found even higher lifetime and 12-months prevalence rates of 16.2% and 6.6%, resp. [3].

### Impairment by MDD

Many studies showed that nearly all patients with MDD suffer from mild to very severe impairment in several domains of life like physical activities, social activities, or occupational responsibilities. E.g., in the above mentioned NCS-R [3] respondents with 12-month MDD reported a mean of 35.2 days in the past year in which they were totally unable to work or carry out their normal activities because of their depression. Following analyses of the World Health Organisation (WHO), MDD persistently belongs to the most disabling diseases worldwide. In 1990, unipolar depressive disorders were the 4^th ^leading cause of disability adjusted life years (DALYs), a measure reflecting the burden of diseases, injuries, and risk factors based on years of life lost due to premature mortality (YLL) and years of life lived in less than full health (YLD). Unipolar depressive disorders have been projected to be the 2^nd ^leading cause of DALYs by the year 2020. A recent re-analysis confirmed this 4th rank for unipolar depressive disorders for the year 2002 as well as the projection as the 2nd leading cause of DALYs for the year 2030, only exceeded by HIV/AIDS. [[4], and references inside].

### Costs associated with MDD

MDD produces substantial costs through hospital admissions, outpatient care and productivity loss as a result of depression-related morbidity, suicide, and other relevant parameters. For Europe (28 countries with approx. 466 million population) the annual costs for depression have been estimated at € 118 billions in 2004. Direct costs alone totalled € 42 billions, comprised of outpatient care (€ 22 billions), drug cost (€ 9 billion) and hospitalisation (€ 10 billion). Indirect costs were estimated at € 76 billions. This makes depression the most costly brain disorder in Europe [5]. Following recent analyses, the USA (approx. 270 million population) was burdened in the year 2000 by $ 26.1 billion direct costs, $ 5.4 billion suicide-related mortality-costs and $ 51.5 billion workplace costs, resulting in a total of $ 83.1 billion [6].

### Outcome of MDD treatment with current strategies is insufficient and more efficient ADs are not to be expected within the next years

The above mentioned data clearly indicate the utmost importance of effective treatments for MDD. The use of antidepressants for the treatment of MDD is well established. However, effect sizes of currently available antidepressants are rather small than medium [7,8]. Drug-placebo difference in efficacy of antidepressants increases with baseline severity, but remains relatively small even for severely depressed patients [7]. Large effectiveness trials like the Sequenced Treatment Alternative to Relieve Depression (STAR*D) study with representative patient populations clearly show that treatment outcome remains disappointing with remission rates of maximal 37% [[9], and references inside]. Antidepressants vary in side effect profile and safety, but hardly with respect to efficacy: e.g. for the 2^nd ^generation antidepressants like selective serotonin reuptake inhibitors, selective serotonin and noradrenaline reuptake inhibitors and the α2-antagonist mirtazapine differences in efficacy have been reported, but only of small effect size [10,11]. Finally, new developments such as the substance P- and CRH-receptor antagonists have failed to prove antidepressant efficacy and the melatonin agonist agomelatine did not show better efficacy than established antidepressants. Thus, outcomes of currently available strategies for MDD treatment are disappointing and more efficient antidepressants are not expected in the next years. Hence, it is sensible to develop new strategies to increase remission rates in acutely depressed patients by means of the antidepressants we currently have.

### Treatment optimisation in the early course of treatment is not implemented in clinical guidelines

A second unmet need of patients suffering from MDD is a shortening of the time to remission. Common clinical view is that antidepressant response usually appears with a delay of several weeks. This view stems mainly from two sources: first, controlled clinical trials aiming to provide evidence for an antidepressants' efficacy usually compare the active compound with placebo. By comparing mean scores of rating scales as measures for depressive symptomatology using repeated measurement analysis of variance, a significant difference between active treatment and placebo usually is detected from week 2-4 and onward. This statistical time lag has long been misinterpreted as indicating a delayed onset of action of antidepressants. Second, pattern analysis [12,13] have suggested that persistent or "true" drug response occurs mainly in the later course of treatment, i.e., week 3-4, while response occurring in the first two weeks was assumed to be unstable and due to placebo effects. Taken together, these interpretations led to the hypothesis of a delayed action of antidepressants and have had substantial impact on clinical practice. Current international and national guidelines for the treatment of MDD usually do not contain recommendations for optimisation strategies (re-assessment of diagnosis, dose adjustments, change of treatment, etc.) in the early course of treatment. The recommended treatment duration until insufficient outcome can be assumed and treatment should be optimised is between 2-4 weeks [14], 3-4 weeks [15,16], 4-6 weeks [17], or 4-8 weeks [18]. Moreover, the low concordance between guidelines reflects substantial uncertainties in the appropriate duration of treatment until the identification of insufficient outcome and no guideline recommendation is based on prospective trials, but only on retrospective data analyses.

### True drug response can be observed within 14 days of antidepressant treatment

Challenging the idea of a delayed onset of antidepressants' action, there is a substantial body of evidence from many retrospective studies with virtually all groups of antidepressants strongly suggesting that a true drug response can be observed within the first 14 days of treatment. [19-27]. E.g., a recent large meta-analysis [22] with data from 4076 patients randomised to active antidepressants and 3045 patients randomised to placebo showed that verum-treated patients were more likely to experience clinical response by 2 weeks or even 1 week of treatment than placebo-treated patients. A further meta-analysis [21] comprising 3418 placebo- and 5158 verum-treated patients found nearly identical time courses of improvement on placebo and active medication: 60.2% and 61.6%, resp., of the improvement occurred within the first 2 weeks. The differences between drug and placebo were most pronounced during the first 2 weeks of treatment and diminished thereafter.

### Early improvement is predictive for final outcome

The occurrence of improvement of depressive symptoms in the early course of treatment has been identified as being highly predictive for final treatment outcome. Nierenberg and colleagues have reported that >50% of patients who eventually respond to fluoxetine treatment started to improve during the first 2 weeks of treatment and that early non-response to fluoxetine treatment predicted poor 8-weeks outcomes [[19], and references Inside]. Katz and colleagues reported on a randomised controlled trial (RCT) in which patients were treated with the selelctive serotonin reuptake inhibitor paroxetine, the tricyclic antidepressant desipramine or placebo [20]. In this study, early treatment-specific behavioural changes occurred that were not observed in the placebo-group, and these changes were highly predictive of ultimate clinical response to antidepressant therapy. In multiple studies between 1993 and 2007, Stassen and colleagues analysed individual time courses of response in depressed patients treated with various antidepressants [[24], and references inside]. A model-finding study with repeated HAMD17 assessments during a 1-week placebo run-in showed that the observed fluctuations did not exceed 15% of baseline score. In consequence, onset of improvement (which models onset of action) has been defined as a 20% baseline score reduction in accordance with clinical practice in which a 4-point HAMD17 reduction (= 20% for a HAMD17 score of 20) is regarded as clinically relevant. Each of their analyses revealed that patients with improvement during the first 2 weeks (= early improvement) of antidepressant treatment showed substantial response at study endpoint. A recent meta-analysis of 2,848 patients with MDD confirmed previous analyses showing that early improvers were far more likely to become responders than patients without early improvement (pooled OR = 9.25, 95%-CI = 7.79-10.98) [24]. In separate analyses, Szegedi and colleagues examined early improvement in a randomised controlled trial comparing mirtazapine and paroxetine in MDD patients [25]. Improvement (HAMD17 score reduction ≥20%) occurred in a majority of patients within 2 weeks of treatment, and this improvement was a highly sensitive predictor of later stable response (HAMD17 score reduction ≥50% at week 4 and onward) and stable remission (HAMD17 score ≤7 at week 4 and onward) for both drugs. Less than 10% of patients who had not improved after 2 weeks of treatment became stable responders or remitters over the course of the study. Szegedi and colleagues recently extended their research to 41 clinical trials with 6,562 MDD patients treated with mirtazapine, serotonine reuptake inhibitors, tricyclics, venlafaxine and placebo [26]. Again, early improvement predicted stable response and stable remission with high sensitivity (>80% and 87%, resp.). Only 11% and 4.1% of patients, who did not improve within the first 2 weeks, became stable responders or stable remitters, respectively. The finding of early improvement being highly predictive for later outcome resulted in the idea that an effective antidepressant treatment triggers and maintains conditions necessary for recovery from the disorder [24]. It has been suggested that affectively ill patients possess a biological, "resilience"-like component that controls recovery from depression to a major extent. Once triggered, recovery seems to follow - independent of pharmacologic differences of the triggers - a uniform pattern of course. Consequently, the vast majority of patients showing a favourable later outcome experience the respective onset within the first 2 weeks of treatment. Inversely, non-improvement after 2 weeks of treatment seems to indicate that a selected antidepressant did not trigger the resilience-like component and has strongly limited chances to do so, even if continued in the course of treatment. Based on these findings in retrospective analyses, leading experts in this field have repeatedly recommended the outcome evaluation in the early course of treatment as well as the consideration of early symptom changes in clinical decision making [22,24-27].

## Methods/Design

### Trial objectives

The primary objective of the trial is to compare effectiveness of EMC with TAU in the treatment of Major Depressive disorder (MDD) in non-improvers on day 14 (level 1 of The EMC Trial). The secondary objectives of the trial are i) to compare speed of recovery from MDD between EMC and TAU in non-improvers on day 14 (level 1 of The EMC trial), ii) to compare the side effect profile and safety of EMC and TAU in non-improvers on day 14 (level 1 of The EMC trial), iii) to compare changes in quality of life under EMC and TAU in non-improvers on day 14 (level 1 of The EMC trial), iv) to compare effectiveness, speed of recovery from MDD, changes in quality of life and side effect profile in subgroups of improvers on day 14 (levels 2 and 3 of The EMC trial).

### Design

**Figure 1:**
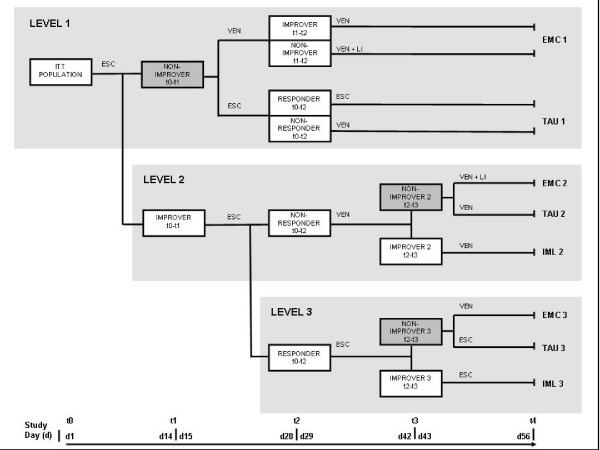
**Design of The EMC Trial**. *Abbreviations: *d = study day; ESC = Escitalopram; LI = Lithium; t = time point; VEN = Venlafaxine; EMC = early medication change group; TAU = treatment as usual group; IML2 = improver group level 2; IML3 = improver group level 3. *Definitions: *improvement = reduction of HAMD17 sum score ≥20% in a specified time span, e.g. between time point (t) 0 and t1; non-improvement = reduction of HAMD17 sum score <20%; response = reduction of HAMD17 sum score ≥50%; non-response = reduction of HAMD17 sum score <20%. Grey boxes indicate randomized groups (non-improver t0-t1; non-improver 2/t2-t3; non-improver 3/t2-t3).

For an overview of the design of the EMC trial, see Figure 1.

#### Level 1

After inclusion and appropriate washout of possible pre-medication with other antidepressants, patients will receive the selective serotonin reuptake inhibitor escitalopram for 14 days. The dose will be escalated to the participant's highest tolerable dose (max. 20 mg/day). Non-improvers, defined by a decrease of <20% on the 17-item Hamilton Depression Rating Scale (HAMD17) [28] between day 0 and day 14 will be randomly assigned to EMC 1 or TAU 1, which both will last 42 days. Patients in the EMC 1 arm will be switched on day 15 to the dual acting selective serotonergic and noradrenergic antidepressant venlafaxine. The dose will be escalated to the participant's highest tolerable dose (max. 375 mg/day). After 14 days of venlafaxine treatment, non-improvers (HAMD17 decrease <20% from day 14-28) will have an augmentation therapy with lithium (plasma level range 0.6-0.8 mmol/l) for 28 days. For improvers under venlafaxine treatment (HAMD17 decrease ≥20% from day 14-28), venlafaxine will be continued for 28 days. Patients randomised to TAU 1 will be treated according to current guidelines, which recommend 28 days of unchanged therapy and use the response criterion (HAMD17 decrease ≥50%) to guide further strategy. Thus, escitalopram will not be changed on day 15, but continued to day 28. In case of response on day 28, escitalopram will be continued for further 28 days. In case of non-response on day 28 (HAMD17 decrease <50%), escitalopram will be switched to venlafaxine (max. 375 mg/day), which will be administered for 28 days.

#### Level 2

Improvers (HAMD17 decrease ≥20%) on day 14 will continue treatment with escitalopram at the highest tolerable dose (max. 20 mg/d). Non-responders on d28 (HAMD17 decrease of <50% between d0-d28) will be treated according to current guidelines and will be switched to venlafaxine. The dose of venlafaxine will be escalated to the participant's highest tolerable dose (max. 375 mg/day). After 14 days of venlafaxine treatment (d29-d42), non-improvers 2 (HAMD17 decrease <20% between d28-42) will be randomly assigned to EMC 2 or TAU 2, which both will last 14 days. Patients in the EMC 2 arm will have an augmentation therapy with lithium (plasma level range 0.6-0.8 mmol/l) for 14 days. Patients randomised to TAU 2 will be treated according to current guidelines, which do not contain recommendations for an optimisation of treatment two weeks after medication switch. Therefore, these patients will continue venlafaxine treatment until day 56. For IML2 under venlafaxine treatment (HAMD17 decrease ≥20% between d28-d42), venlafaxine will be continued for 14 days at the participant's highest tolerable dose.

#### Level 3

Responders on d28 (HAMD17 decrease of ≥50% between d0-28) will continue treatment with escitalopram at the highest tolerable dose. Remitters on d28 (HAMD17 sum score ≤7) will be regarded as responders. After further 14 days of escitalopram treatment (d29-42), non-improvers 3 (HAMD17 decrease <20% between d28-42) will be randomly assigned to EMC 3 or TAU 3, which both will last 14 days. Remitters on d42 (HAMD17 sum score ≤7) will be regarded as improvers and will not be randomized. Patients in the EMC 3 arm will be switched to venlafaxine. The dose of venlafaxine will be escalated to the participant's highest tolerable dose (max. 375 mg/day). Patients randomised to TAU 3 will be treated according to current guidelines, which do not contain recommendations for an optimisation of treatment in case of non-improvement after an initial response. For IML3 with continued escitalopram treatment (HAMD17 decrease ≥20% between d28-42), escitalopram will be continued for 14 days at the participant's highest tolerable dose.

### Trial duration

The duration of this trial is expected to be 36 months. The subject recruitment started in September 2009; the end of the recruitment period is planned for February 2012. The actual overall trial duration or subject recruitment period may vary from this time period.

### Primary endpoint

Remission from MDD, defined as a HAMD17 sum score ≤7 [29] on day 56, is the primary endpoint and will be analyzed only for non-improvers on day 14 (level 1 of the EMC Trial). Remission denominates the complete absence of residual clinical symptoms and functional impairments. It is well established that symptomatic response without remission is associated with a worse prognosis and continuing functional disability. For these reasons, acute-phase treatment aims at symptom remission, not just response. Remission from MDD has become the most important outcome parameter in trials investigating the effectiveness of interventions in patients with MDD. The HAMD17 scale is one of the most widely used clinician rated instruments for the assessment of mood disturbances, has been internationally validated and proven to be sensitive to change.

### Secondary endpoints

Response, defined as a HAMD17 sum score decrease ≥50% on day 56. Response is generally regarded as a clinically relevant treatment outcome and commonly used in clinical trials. A reduction of ≥50% from baseline score is the usual definition. Furthermore, we will asses the following secondary endpoints: absolute change of HAMD17 sum score between d0 and d56; remission and response, defined as a sum score ≤11 on the 30-item Inventory of Depressive Symptomatology (IDS-C30) and 50% baseline score reduction, respectively, on day 56. The IDS is available in the clinician (IDS-C30) and the self-rated version (IDS-SR30); remission is defined as a sum score ≤11, response as a 50% baseline score reduction. The IDS-C30 is sensitive to changes in depressive severity; evidence of acceptable psychometric properties of the IDS scales and correlations with other established rating scales (e.g., HAMD17) are available http://www.ids-qids.org. The self-report version was developed to be an "easy to use" severity measure, providing a potentially more time efficient alternative to clinician rated instruments in both clinical or research settings.

In levels 2 and 3 of the EMC trial, further secondary endpoints will be assessed: time to remission and time to response according to IDS and HAMD17; remission from MDD, defined as HAMD17 sum score ≤ 7 on day 56 (in subgroups of improvers on day 14 entering level 2 or level 3 of The EMC trial); absolute change in SF-12 subscales "physical component score" and "mental component score" (day 56-day 0); occurrence of adverse events, UKU ratings [30] at all visits and relevant laboratory data (routine laboratory, therapeutic drug monitoring).

### Selection and withdrawal of subjects

In order to acquire a sample representative of inpatients with MDD, the EMC trial has broad inclusion criteria that allow enrolment of both adult and elderly patients, moderately to very severely depressed patients as well as MDD patients with other psychiatric comorbid disorders. The EMC trial prospectively enrols inpatients of the participating centres, which assures the recruitment of help-seeking patients and provides highest adherence and compliance to protocol treatment. Hospitalisation of patients can occur after referral from the treating general practitioner, from the treating psychiatrist in the outpatients sector, from another hospital (e.g. after treatment in an intensive care unit in case of suicide attempt) or immediately after consultation of the departments' physician in charge. In general, the indication for emergent hospitalisation in a department of psychiatry is given in case of acute suicidality or risk of acute endangerment for others as well as in case of prominent psychotic symptoms. Typical indications for hospitalisation of patients with Major Depression are a severity of depression exceeding the capacity of outpatient care including suicidality, the risk of isolation due to depression and other severe psychosocial factors, life circumstances, which significantly impair the treatment success, resistance to outpatient treatment, high risk for (further) transition into chronicity of the disease. No subject will be allowed to be enrolled in this trial more than once.

#### Inclusion criteria

Subjects meeting all of the following criteria will be considered for admission to the trial:

• Major Depressive Disorder (MDD), first episode or recurrent, according to DSM-IV

• A HAMD17 score of ≥18 pts. Although there are different cut-offs for mild to moderate depression varying between 13/14-17/18 pts., the cut-off of ≥18 pts. is the most often used in clinical trials and assures the inclusion of at least moderately depressed patients.

• Age between 18 and 65 years and age ≤ 60 years at the time of the first depressive episode. This age range assures the inclusion of patients with early-onset depression in contrast to late-onset depression, for which different pathophysiological mechanisms have been suggested. In addition, the vast majority of studies on the predictive value of early improvement included patients aged ≤ 65 years.

• Ability of subject to understand character and individual consequences of clinical trial.

• Signed and dated informed consent of the subject must be available before start of any specific trial procedures.

#### Exclusion criteria

Subjects presenting with any of the following criteria will not be included in the trial:

• Acute risk of suicide needing an intervention not comprised by protocol treatment (e.g. electroconvulsive therapy)

• Patients with a lifetime DSM-IV diagnosis of dementia, schizophrenia, schizoaffective disorder, bipolar disorder (this group is excluded because they have a psychiatric condition that requires a different treatment).

• Patients with a current DSM-IV diagnosis of posttraumatic stress disorder, obsessive-compulsive disorder, anxiety disorder, or eating disorder and the requirement of a treatment not comprised by protocol treatment.

• Patients with DSM-IV substance dependency requiring acute detoxification.

• Depression due to organic brain disorder, e.g. Multiple Sclerosis and Parkinson's Disease

• Women who are pregnant, breastfeeding or planning to become pregnant during the trial (a pregnancy test will be performed at screening visit)

• Women who are not sterile by surgery or for more than two years postmenopausal or women with childbearing potential who not practicing a medically accepted contraception during trial (reliable contraception are systemic contraceptives (oral, implant, injection), diaphragm or condoms with spermicide, sexual abstinence).

• Patients currently taking antidepressant medication, which has been started within the 2-4 weeks prior to study, begin and a continuation of this antidepressant medication is clinically indicated.

• A clear history of non-response to an adequate treatment trial in the current major depressive episode to any protocol antidepressant. A "clear history of non-response" has to be assumed, when the following criteria are fulfilled:

◦ ad Escitalopram: Treatment with a mDDD ≥15 mg/d for 4 weeks or C_PL _15-80 ng/ml for four weeks without response, i.e. a symptom reduction ≥50% between start and end of treatment.

◦ ad Venlafaxine: Treatment with a mDDD ≥300 mg/d for 4 weeks or C_PL _195-400 ng/ml for four weeks without response, i.e. a symptom reduction ≥50% between start and end of treatment;

◦ ad Lithium: Treatment with C_PL _0.6-0.8 mol/l Li^+ ^for four weeks without response, i.e. a symptom reduction ≥50% between start and end of treatment.

• History of medical or psychological condition, metabolic dysfunction, physical examination finding, or clinical laboratory finding giving reasonable suspicion of a disease or condition that contraindicates the use of an investigational drug or render the patient at high risk from treatment complications.

• History of hypersensitivity to the investigational medicinal product or to any drug with similar chemical structure or to any excipient present in the pharmaceutical form of the investigational medicinal product.

• Clinically significant or unstable medical or surgical condition that may preclude safe and complete study participation. Such conditions may include gastrointestinal, cardiovascular, vascular disease, pulmonary/respiratory, hepatic impairment, renal, metabolic diseases, endocrinological, neurological, immune-deficiency, haematopoietic disease, or malignancies as determined by medical history, physical examination, or laboratory tests.

• Participation in other clinical trials during the present clinical trial or within the last 6 months.

• Medical or psychological condition that would not permit signing of informed consent.

#### Withdrawal criteria

Subjects can be withdrawn their consent at their own request without given reasons at all time during the trial. This will be without disadvantages for the subject. However, the investigator should try to perform a final visit to get concluding findings of investigation. Participants withdrawn from the study will not be replaced, regardless of the reason for withdrawal. The participation in this study may be discontinued due to any of the following reasons: i) at their own request; ii) for safety reasons at the request of the Sponsor; iii) request of a regulatory agency; iv) significant adverse events related to the therapy (participants will be followed up for treatment response and safety); v) participant is non-compliant or not sufficiently compliant with the study procedures/study protocol; vi) if, in the investigator's opinion, continuation of the trial would be detrimental to the subject's well-being; vii) participant needs a medication not allowed in the protocol during the study; viii) any clinically significant change in participant's pre-study medical condition; ix) positive pregnancy test (safety follow-up up to and including the premature termination of the pregnancy or delivery of the child and assessments of effects on the foetus or child). The Investigator decides about withdrawal of subjects from the clinical trial in case of occurrence of criteria mentioned above. In all cases, the reason for withdrawal must be recorded in the case record file and in the subject's medical records. In case of withdrawal of a subject at his/her own request, as far as possible the reason should be asked for and documented. The subject must be followed up and as far as possible all examinations scheduled for the final trial day should be performed and documented. All ongoing serious adverse events of withdrawn subjects have to be followed up until no more signs and symptoms are verifiable or the health condition of the subject has stabilized, but no longer than 6 months after subjects discontinuation from the trial.

### Measures taken to minimize/avoid bias

#### Randomisation

Randomisation lists stratified by level (1 to 3) and centre will be generated by the IZKS. The randomisation ratio will be 1:1 using block randomisation. Randomisation is not stratified by severity of depressive symptoms or duration of the disease. The very close relation between early non-improvement and final treatment failure during antidepressant treatment has been identified in several clinical studies covering a broad range of severities and durations of depression (see introduction section). We identified the same relation in patients with mild major, minor or subsyndromal depression (Tadić et al. 2010). Taken together, the currently available data support the idea that early non-improvement is a general marker of inefficacy of antidepressant treatment and that this very close relation between early non-improvement and final failure of treatment response exists independently of severity or duration of depression. Centre effects are not to be expected; in the STAR*D trial [[9], and refs. Inside] remission and response rates did not differ between 18 primary and 23 psychiatric care settings. Nevertheless, centre effects will be analysed post hoc. A web based randomisation tool developed by IZKS Mainz will be used within this trial allowing investigators to randomize patients via a secure web interface. Role specific access rights and the need to confirm all details necessary for stratified randomisation (level and centre) are incorporated within the tool and will reduce the risk of misuse and unintended randomisations. The randomisation tool "e-randomiXer" has been released for use within the study after all components implemented specifically for The EMC Trial have been sufficiently tested.

#### Blinding

To prevent assessment bias, trained raters will be blinded to treatment. The training was carried out using five videotaped interviews using the HAMD17 and three interviews using the IDS-C30. All patients had given informed, written and videotaped consent prior to the interview. Thirteen raters from six trial sites were trained. Prior to the training the video tapes were rated by two experienced raters. Rating agreement (accuracy) of the individual items of HAMD17 and IDS-C30 with the expert ratings as well as the intra class correlation (ICC) were high (detailed results will be presented elsewhere).

### Treatments, dosage schedule and rationale for dose selection

The EMC Trial will use only established drugs in the treatment of MDD. For the antidepressants escitalopram and venlafaxine an equal efficacy in the acute-phase treatment of MDD has been reported [31]. Both escitalopram and venlafaxine will be escalated to the participant's highest tolerable dose, which (1) allows clinicians to delay dose increases if the participant is initially intolerant; (2) approximates clinical practice; (3) allows some initially intolerant participants to accommodate to a particular dose, subsequently permitting a dose increase with an acceptable side effect burden; and (4) allows participants with concurrent general medical conditions to be retained in the trial while also receiving adequate doses.

#### Escitalopram

Escitalopram is a selective serotonin reuptake inhibitor and is licensed for the treatment of major depressive episodes by national and international federal drug agencies worldwide. In the treatment of MDD, escitalopram is an established first choice drug. There is evidence suggesting a statistically significant difference favouring escitalopram over other antidepressants [10]. A fixed-dose-study showed a slightly better outcome in patients with 20 mg escitalopram/d compared to patients treated with 10 mg/d, suggesting a higher efficacy of 20 mg/d, particularly in patients with severe depression [32]. In order to achieve maximal effectiveness, escitalopram will be titrated to 20 mg/d according to its German federal license for the treatment of MDD. Escitalopram should be administered at 10 mg on day 1 and at 20 mg on day 2 and onwards. Escitalopram should be administered in the morning and once daily. Dose adjustments, e.g. dose reduction in case of side effects, are allowed.

#### Venlafaxine

Venlafaxine is a selective serotonin reuptake inhibitor at 75 mg/d and dual acting antidepressant (norepinephrine and serotonin transporter inhibition) at doses of 150 mg and above. At higher doses it also inhibits dopamine reuptake. It is licensed by national and international federal drug agencies worldwide for the treatment of major depressive episodes. The results of numerous meta-analyses of the efficacy data for venlafaxine support the conclusion that venlafaxine is a superior antidepressant [33]. The switch from selective serotonin reuptake inhibitor to venlafaxine after treatment failure is supported by a meta-analysis of 3 randomised controlled trials showing the superiority of the switch to venlafaxine versus another selective serotonin reuptake inhibitor [34]. Available dose finding studies showed a positive dose-response relationship for venlafaxine in the treatment of MDD with a superior efficacy at 375 mg/d compared to 225 mg/d, 150 mg/d, 75 mg/d, and placebo [35]. In order to achieve maximum effectiveness, venlafaxine will be titrated to max. 375 mg/d according to its German federal license for the treatment of MDD. Venlafaxine should be administered at 75 mg on day 1, at 150 mg on day 2, at 300 mg on day 3 and at 375 mg on day 4. Venlafaxine should be administered in the morning and once or twice daily. Dose adjustments, e.g. dose reduction in case of side effects, are allowed.

#### Lithium augmentation

Lithium augmentation is by far the most often studied strategy in depressed patients with insufficient response to an antidepressant (27 open and 10 randomised placebo-controlled trials). A recent meta-analysis of these 10 randomised controlled trials showed that lithium had a positive effect versus placebo, with an odds ratio of 3.11, which corresponds to a number needed to treat of 5. The mean response rate was 41.2% in the lithium group and 14.4% in the placebo group (p < .001) [36]. In the EMC trial lithium dose will be strictly adjusted according to the established plasma level range of 0.6-0.8 mmol/l for the treatment of MDD [15,37]. Lithium should be administered at 12 mmol on day 1 and at 24 mmol on day 2. Lithium can be administered in the morning or in the evening and once or twice daily. Dose adjustments, e.g. dose reduction in case of side effects, are allowed. After 1 week of lithium treatment, plasma concentration will be determined weekly. Dose adjustments will be performed to reach the target plasma concentration of 0.6-0.8 mmol/l.

### Concurrent medication

In general, the principles guiding the prohibition and permission of additional treatments try to balance the need to minimise the confounding effects of treatments that may alter the effect sizes of treatments under study with the goals of increasing the feasibility of conducting a randomised treatment protocol and of increasing treatment adherence among participants. By allowing many additional treatments that are widely used in practice, the EMC Trial enhances both generalisability and feasibility. Additional pharmacotherapy will be allowed to treat transient associated symptoms (e.g., insomnia) or transient medication side effects (e.g., agitation or anxiety); i.e. short-acting sedatives (zolpidem or zopiclone), the low potency neuroleptic drug pipamperone, the histamine-receptor antagonist promethazine in standard doses as well as benzodiazepines in a dose-equivalent to max. 15 mg of diazepam. Participants may receive treatment for concurrent general medical conditions, as long as these medications do not contraindicate the use of protocol medications. Any antidepressant medication taken at study entry must be discontinued (after consent) before beginning escitalopram. Appropriate wash-out will be assured by determination of plasma drug concentration. Not permitted drugs for the entire duration of the EMC trial are drugs that are contraindicated during the treatment with one of the three protocol medications. These drugs are: i) irreversible non-selective inhibitors of the monoamino-oxidase (MAO); ii) the reversible, selective MAO-A inhibitor moclobemide; iii) the reversible, non-selective MAO-inhibitor linezolide; iv) the irreversible, selective MAO-B-inhibitor selegiline; v) other antidepressant drugs; vi) antipsychotic drugs; vii) mood-stabilizing agents other than lithium. Furthermore, not permitted drugs for the entire duration of the EMC Trial are drugs with substantial influence of the major endpoints of the study.

### Procedures for monitoring subject compliance

Trial medication will be dispensed to the subjects by the investigator. During hospitalisation the medication will be distributed by a study nurse and the patient's compliance will be assessed by counting of unused tablets. Additionally, compliance will be assessed by therapeutic drug monitoring (TDM). In case of out-patient treatment (i.e., in case of demission from hospital during the study), subjects will be instructed to bring all trial medication to the trial site at every visit (including all empty packages and unused trial medication). Compliance will be assessed by counting of unused tablets and TDM.

### Measurements/Trail Schedule

**Figure 2:**
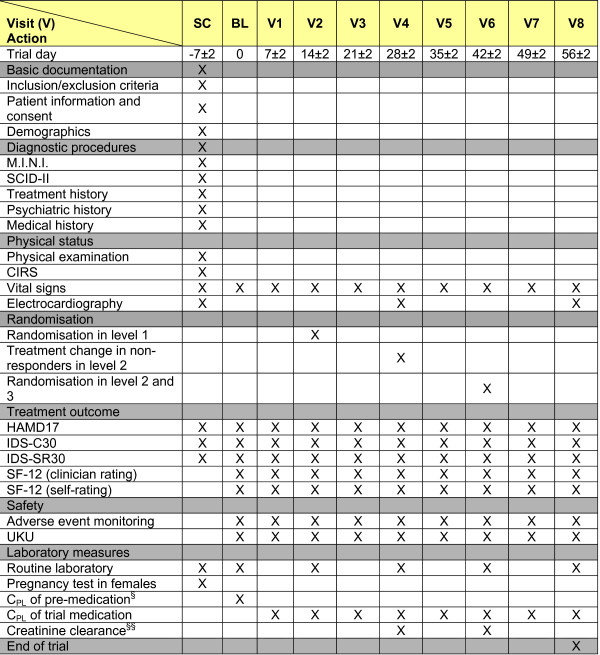
**Schedule of The EMC Trial**. ^§ ^in case of existing pre-medication to assure complete wash-out; ^§§ ^in case of lithium treatment; Abbreviations: BL (baseline visit); CIRS: Cumulative illness Rating Scale; CPL (plasma concentration); HAMD17 (17-item Hamilton-Depression-Rating-Scale); IDS-C30 (30-item Inventory of Depressive Symptomatology); SC (screening visit); M.I.N.I. (Mini International Neuropsychiatric Interview); SCID-II (Structured Clinical Interview for DSM-IV Axis II Disorders); SF-12 (12-item Short Form Health Survey); UKU (Udvalg for Kliniske Undersogelser).

For an overview of the measurements of the EMC Trial, see the trial schedule (Figure 2).

#### Assessment of efficacy

Assessment of efficacy comprises symptomatic and functional changes from screening or baseline to study end in weekly intervals. Symptom changes will be assessed by the HAMD17. Additionally, depression severity will be assessed with the 30-items clinician-rated version of the Inventory of Depressive Symptomatology (IDS-C30). These interviews will be performed by trained raters. The IDS is also available as a self-rating instrument (IDS-SR30). It will be used to evaluate the patients' perspective of symptom severity. Patients' function will be assessed using the Short-Form Health Survey as a measure for health-related quality of life independent of psychiatric diagnosis. Its 12-item version assesses the two dimensions "physical health" and "psychic health" as subscales. Each item refers to a different symptom concerning either "physical health" or "psychic health". The interview will be performed by trained raters. Raters will be blind to randomization in order to prevent assessment bias. The Short-Form Health Survey is also available as a self-rating instrument. It will be used to evaluate the patients' perspective of impaired function.

#### Assessment of safety

##### Definitions

Adverse events (AE), serious adverse events (SAE), and serious adverse reactions (SAR) as well as suspected unexpected serious adverse reactions (SUSAR) are defined are according to the Guideline for Good Clinical Practice of the International Conference on Harmonisation (ICH-GCP) in its current version (CPMP/ICH/135/95/Step 5, ICH Topic E6 (R1); http://www.ema.europe.eu).

##### Assessment of AEs by the investigator

AEs will be assessed by the investigator in weekly intervals (see figure 2). Additionally, inpatients will receive daily clinical visits by the ward physician; in case of a new symptom the investigator will be contacted in order to decide whether the new symptom fulfils the criteria of an AE, SAE, SAR or SUSAR. Adverse events will be assessed in terms of their seriousness, severity, and relationship to the study drug.

##### Period of observation

In this trial, the period of observation for collection of adverse events extends from the time the subject has signed the informed consent document up to study day 56 (end of the trial). All subjects who have adverse events, whether considered associated with the use of the investigational products or not, must be monitored to determine the outcome. The clinical course of the adverse event will be followed up according to accepted standards of medical practice, even after the end of the period of observation, until a satisfactory explanation is found or the investigator considers it medically justifiable to terminate follow-up, but no longer than 30 days after the end of the trial. Should the adverse event result in death, a full pathologist's report should be supplied, if possible. If the investigator detects a serious adverse event in a trial subject after the end of the period of observation, and considers the event possibly related to the prior trial, he should contact the sponsor to determine how the adverse event should be documented and reported.

##### Documentation of AEs

All AEs (whether serious or non-serious) reported by the subject or detected by the investigator will be documented on the "Adverse Event" pages of the CRF. AEs will also be documented in the subject's medical record. If the adverse event is serious (see below), the investigator must complete, in addition to the "Adverse Event" page, a "Serious Adverse Event" form in the ISF at the time the SAE is detected. This form must be immediately sent to responsible SAE Management of the independent Interdisciplinary Centre for Clinical Trials (IZKS) at the University Medical Center Mainz. Every attempt will be made to describe the adverse event in terms of a diagnosis. If a clear diagnosis has been made, individual signs and symptoms will not be recorded unless they represent atypical or extreme manifestations of the diagnosis, in which case they should be reported as separate events. If a clear diagnosis cannot be established, each sign and symptom must be recorded individually.

##### Immediate reporting by investigator

SAEs must be reported immediately within 24 hours after the SAE becomes known to IZKS Mainz. The initial SAE Report must be as complete as possible including details of subject's identification (screening number, random number), the (serious) adverse event (medical term, diagnosis), the trial medication and an assessment of the causal relationship between the event and the trial medication made by the investigator. The investigator should provide related additional information on the clinical course and the outcome of each SAE as soon as possible to IZKS Mainz using the SAE form (Follow up report). In addition, any pregnancy diagnosed in a female subject or in the female partner of a male subject during treatment with the investigational product must be reported to the sponsor immediately via facsimile using the SAE form. Worsening of a sign or symptom of the condition under treatment will normally be measured by efficacy parameters. However, if the outcome fulfils the definition of "serious adverse event", it must be reported as such.

##### Safety evaluation by sponsor

According to GCP the sponsor is responsible for the continuous safety evaluation of the investigational product(s) and the clinical trial. On behalf of the sponsor, IZKS Mainz will conduct the management of SAEs and the expedited reporting as required by German Drug Law (AMG) and GCP regulation (GCP-V). Suspected unexpected serious adverse reactions (SUSARs) and safety issues as defined by GCP-V are determined for expedited reporting: The competent authorities and the ethics committees should be notified as soon as possible but not later than 15 calendar days if the event is non-fatal and 7 calendar days if it was fatal. All investigators should be informed too. Work flow and procedures concerning SAE management will be described in a safety manual. During the clinical trial the sponsor will submit the annual safety report including a list of all serious adverse reactions to the ethics committee(s) and the competent authorities once a year. A Data and Safety Monitoring Committee (DSMC) is established to supervise the clinical trial (see below).

##### Other safety data

All observations pertinent to the safety of the study medication will be recorded on the CRF and included in the final report. Safety variables are as follows: UKU scale, laboratory changes (panels for electrolytes, creatinine, liver enzymes, haematology, creatinine, creatinine clearance in case of lithium treatment), changes in vital signs (blood pressure, heart rate and temperature) and, cardiologic evaluation by ECG. The UKU scale and recording of vital signs will be performed in weekly intervals; routine laboratory will be collected in bi-weekly intervals; ECG will be performed in four-week intervals.

### Statistics

Details of the statistical analysis of the data collected in this trial will be documented in a Statistical Analysis Plan (SAP) that will be generated by IZKS Mainz and finalized before closing the data base. The SAP is based on the protocol including all amendments. The document may modify the plans outlined in this protocol; however any major modifications of the primary endpoint definition and/or its analysis will also be reflected in a protocol amendment. Any deviation from the original statistical plan will be described and justified in the final report. The statistical analysis will be conducted by means of SAS^®^.

#### Sample size

The sample size has been calculated for level 1 of the EMC Trial (see Fig. 1 for trial design). For TAU 1, non-improvers on day 14 continuing medication are expected to become non-responders on day 28 in about 88% and responders in 12% [25]. The switch from escitalopram to venlafaxine led to a remission in 24% after 14 weeks of treatment [38]; for the treatment period of this trial (day 29-56), we assume a remission rate of 20% on day 56 for TAU 1. Responders on day 28 continuing medication are expected to become remitters on day 56 in 75% [25], resulting in an expected overall remission rate of 26% for TAU 1. In the EMC 1 arm, non-improvers on day 14 switched to venlafaxine are expected to show improvement in 70% and non-improvement in 30% [24,25] after 14 days of treatment, i.e. on study day 28. Improvers after 14 days (i.e. study day 28) continuing venlafaxine medication are estimated to become remitters in 55% [24,25]; lithium augmentation in non-improvers on study day 28 is expected to lead to a remission rate of 34% [39]. Taken together, an overall remission rate of 49% on day 56 is estimated for EMC 1. A drop out rate of 12% is assumed; drop-outs will be classified according to the last available HAMD17 sum score whereby HAMD17 scores before day 28 will not be carried forward. Patients with no HAMD17 score available from day 28 until day 56 will be considered as non-remitters. Hence, remission rates of 49% and 26% in the underlying population correspond to 43% and 23% if 12% of dropouts are counted as non-remitters. These proportions result in an odds ratio of 2.5 and require sample sizes of 96 patients per group (alpha = 0.05, Fisher's exact test, 2-sided) for a power of 80%. Therefore, it is planned to randomise 192 patients of non-improvers on day 14. As of 30% of patients enrolled in the study are expected to be non-improvers after 14 days of escitalopram treatment, 640 patients (= ITT sample) have to be enrolled in the study and 1280 patients must be screened under the assumption that 50% are willing to participate.

#### Analysis populations

All subjects who signed informed consent are considered as enrolled subjects, even if they did not receive any trial treatment. The Intention-to-treat (ITT) population comprises all enrolled subjects. Within ITT population analyses, subjects will be assigned to the treatment to which they were randomised. To be eligible for the per protocol (PP) population, subjects must fulfil the following criteria: i) all visits have been performed; ii) each visit has been performed according to visit schedule; iii) treatment compliance of at least 90% measured by drug accountability. Additional criteria will be defined during the conduct of the trial and before database closure. The safety population comprises all subjects who received at least one dose of trial treatment. In analyses of the safety population, subjects will be assigned to the treatment which they actually received. The analysis populations will be defined prior to the database closure. Within the ITT and PP population the following sub-collectives will be defined (see also figure 1):

##### Level 1

• Non-improvers on day 14 = EMC1 + TAU1. These patients of the ITT sample represent the analysis population of the primary endpoint.

##### Level 2

• Improvers on day 14, who are non-responders on day 28 and non-improvers on d42 = EMC 2 + TAU 2

• Improvers on day 14, who are non-responders on day 28 and have venlafaxine treatment from day 43 to endpoint = TAU 2 + IML 2

##### Level 3

• Responders on day 28, who are non-improvers on day 42 = EMC 3 + TAU 3. Remitters on d42 will be counted as improvers regardless of the HAMD17 total score change from d28-42.

• Responders on day 28, who have escitalopram treatment from day 43 to endpoint = TAU 3 + IML 3

##### Other

• Dropouts before a possible randomisation

#### Efficacy analyses

The primary analysis population of the primary endpoint is the ITT Population of non-improvers on day 14 (level 1 of the EMC trial). All hypotheses will be tested on a two-sided level of significance α = 0.05.

#### Analysis of primary endpoint

The primary endpoint is remission from MDD, defined as a HAMD17 sum score ≤ 7, on day 56 in non-improvers on day 14. The null hypothesis to be tested is:

H_0_: RRD56_EMC _= RRD56_TAU _vs. H_a_: RRD56_EMC _≠ RRD56_TAU_

RRD56 denotes the remission rates on day 56 in the respective treatment group. It is assumed that remission rates are higher in the EMC 1 group. The hypothesis will be tested by Fisher's exact test using a two-sided significance level of 0.05. Additionally a 95% confidence interval for the difference in remission rates will be calculated. In the primary analysis HAMD17 sum scores will be carried forward from day 28 on. Patients with no HAMD score available between day 28 and 56 will be classified as non-remitters. This approach is rather conservative; however, in our opinion it is justified, because all of these patients are non-improvers at week 2 and it is likely that these patients would have become final non-remitters after being non-improver at week 2. Missing data patterns are not expected to differ between treatment groups and no bias is expected due to this approach. There are quite a few methods for multiple imputation available; the choice of the appropriate method depends on several factors including the missing value pattern. Here, we prefer a method for replacing missing data that can be unambiguously specified in advance which is essential in confirmatory trials. Nevertheless, we will incorporate an analysis imputing missing values multiply as a further sensitivity analysis. The analysis of the primary endpoint will be repeated for the Per-Protocol-Population comprising all non-improvers on day 14 with a minimum of adherence to the trial protocol in a descriptive way. As a secondary analysis, a logistic regression of remission on treatment group, trial site, sex, baseline severity of depressive symptoms, comorbid disorders, plasma concentrations of ADs and additional treatments will be performed.

#### Analysis of secondary endpoints

Remission according to HAMD17 (definition see primary endpoint) in all ITT and PP sub-collectives of level 2 and 3.

The following secondary endpoints will be analyzed separately for the ITT and PP sub-collectives of level 1, 2 and 3 defined above:

• The comparison of response rates to AD treatment defined as HAMD17 sum score decrease of at least 50% on day 56, and remission rates according to IDS will be analysed in the same way as the primary endpoint.

• Absolute change in HAMD17 sum scores will be analyzed using a two sample t-test.

• Remission/response according to IDS will be analyzed in the same way as the corresponding HAMD17 analyses.

• Comparison of time to remission and time to response will be analyzed by means of life table estimates.

• Absolute change in SF-12 scores will be analyzed using a two sample t-test.

All analyses of secondary endpoints will be interpreted exploratory. Corresponding 95% confidence intervals for the estimated parameters will be calculated. Descriptive statistics will be displayed for all parameters.

#### Analysis of adverse events

All summaries and listings of safety data will be performed for the safety population. Frequencies of subjects experiencing at least one adverse event (AE) will be displayed by body system and preferred term according to MedDRA terminology. Detailed information collected for each AE will include: A description of the event, duration, whether the AE was serious, intensity, relationship to trial drug, action taken, clinical outcome. Summary tables will present the number of subjects observed with AEs and corresponding percentages. Additional subcategories will be based on event intensity and relationship to trial drug. A subject listing of all AEs will be prepared. UKU ratings collected at every visit starting at baseline will be presented in a way that changes in the course of the study can be identified.

#### Analysis of clinical laboratory findings, ECG values and vital signs

Listings will be prepared for each laboratory measure and will be structured to permit review of the data per subject as they progress on treatment. Summary tables will be prepared to examine the changes of laboratory measures over time. Additionally, shift tables will be provided to examine the changes of laboratory data from normal baseline to values outside the corresponding reference range during/after treatment.

### Ethical aspects

#### Good clinical practice

The procedures set out in this trial protocol, pertaining to the conduct, evaluation, and documentation of this trial, are designed to ensure that all persons involved in the trial abide by good clinical practice (GCP) and the ethical principles described in the Declaration of Helsinki. The trial will be carried out in accordance with local legal and regulatory requirements. The requirements of the AMG, the GCP regulation, and the Federal Data Protection Law (BDSG) will be kept. The trial was approved in July 2009 by the ethics committee at the Landesärztekammer Rheinland-Pfalz (code: 837.211.09 (6717)).

#### Summarized risk-benefit assessment

All drugs used within the EMC Trial are established in the treatment of major depression and licensed by national and international federal drug agencies worldwide for the treatment of major depression. Relying on current treatment guidelines, it is standard care to continue treatment in patients without improvement in the early course of treatment. This strategy has to be questioned in the light of the substantial data basis supporting the view that non-improvement predicts poor later outcome. Thus, the EMC trial will provide answers to this important question.

#### Data and Safety Monitoring Committee (DSMC)

The trial is supervised by a data and safety monitoring committee that consists of one Chair of a University Department of Psychiatry in Germany with substantial experience in clinical trials, one clinical pharmacologist with special experience in pharmacovigilance and one biometrician, who is head of an University Institute of Medical Statistics, Informatics and Epidemiology in Germany. This DSMC supervises the progress of the trial, monitors the safety data, reviews all relevant information on the trial topic from all other sources, ensures adherence to the protocol, advices whether to continue, modify or stop the trial and provides the funding organisations with information and advice in accordance with the DSMC Standard Operating Procedure. The DSMC meetings will be held once a year.

## Discussion

The EMC trial is unique because it investigates for the first time prospectively whether MDD patients with non-improvement after 14 days of AD treatment with EMC are more likely to become remitters compared to patients treated according to current guidelines, i.e., with a medication change after 28 days of treatment in case of non-response. All current evidences originate from retrospective data analysis, but not from prospective clinical trials: there is substantial evidence showing that non-improvement in the first 2 weeks of AD treatment is highly predictive for poor final outcome and leading experts in the field have repeatedly recommended the outcome evaluation in the early course of treatment as well as the consideration of early symptom changes in clinical decision making [23-27]. However, the entire knowledge about the predictive value of early improvement derives exclusively from retrospective data analyses. No study has yet prospectively investigated whether non-improvers (HAMD17 decrease <20%) after 14 days of AD treatment with EMC are more likely to become remitters compared to patients with a medication change after 28 treatment days in case of non-response (HAMD17 decrease <50%). Thus, the EMC trial is indispensable to prove the clinical value of early improvement and to realise the transfer from retrospective analyses to convincing prospective clinical trials. If our hypothesis holds true that the EMC strategy leads to a relevantly higher proportion of remitted patients, this could have a substantial impact on various aspects of MD treatment, e.g. patients' welfare, clinical guidelines, daily clinical practice, economic costs of MD as well as research settings.

The protocol of the EMC trial schedules in level 1 a randomization step for non-improvers two weeks after treatment start. This approach focuses on a patient group that is at high risk for treatment failure. The same approach has been applied in levels 2 and 3 of the EMC trial, where randomization steps are scheduled for patients who experience no further symptom amelioration after an initial improvement or response, respectively. This approach represents an advantage compared to other possible designs such as randomising all participants at baseline to TAU vs. EMC. Such a design would be inappropriate, because it would randomize patients with a favourable treatment outcome to the first antidepressant medication to both EMC and TAU. There is no need for the evaluation of an EMC strategy in patients with a continuous and favourable treatment outcome, because there is no indication for a treatment change in these patients. Furthermore, such a design would erroneously diminish the effect size of the EMC strategy compared to TAU.

The design of the EMC trial schedules as primary outcome "remission at day 56" after treatment start. This is a common duration in antidepressant treatment trials. However, after an initial improvement the continuous amelioration of symptoms and finally the development of remission might exceed the time period of 6 weeks (e.g. after the start of venlafaxine treatment on day 15 in EMC 1), or 4 weeks (e.g. after the augmentation of venlafaxine with lithium in EMC 1 or after the start of venlafaxine treatment in TAU 1). Therefore, the remission rates obtained in subgroups of the EMC trial might be lower compared to a design that would schedule a primary endpoint at week 10 or 12 weeks after treatment start. However, this applies to both EMC and TAU strategy and there is no reason to expect differences in the speed of the development of remission after an initial improvement between groups. Therefore, the design of the EMC trial is economic and meaningful with regard to the primary efficacy measure "remission".

Very long treatment duration requires substantial patience and adherence from depressed patients, which is particularly difficult, when pessimism and hopelessness dominate the outlook of these patients. Ineffective therapy is especially problematic in MD because it can increase the risk that patients lose confidence in and detach from their treating physicians, stop taking their prescribed medication, or lose hope that their symptoms can be effectively treated. As a result, the risk of serious complications, such as suicide, is increased.

Current national and international clinical guidelines usually do not contain recommendations for adapting an individual's treatment during the early course of therapy, but schedule a treatment duration between 2-4 weeks [14], 3-4 weeks [15,16], 4-6 weeks [17], 4-8 weeks [18] until insufficient outcome is diagnosed and treatment optimisation is recommended. Furthermore, recommendations for the optimal duration of treatment until identification of insufficient outcome vary from guideline to guideline and are not based on prospective clinical trial evaluating the effectiveness of different treatment strategies, but on retrospective data analysis reflecting the assumption of a delayed onset hypothesis. On the other hand, acknowledging the growing data basis that non-improvement is highly predictive for final treatment outcome, more recent guidelines [14-16] have already shortened the time span until treatment optimisation in case of insufficient outcome compared to older ones [17,18]. However, although significant data basis suggests that treatment failure can be already predicted after two weeks, guidelines have not yet included a regularly treatment optimization after two weeks. This is entirely justified because of the lack of a prospective clinical trial. The EMC Trial will close this significant gap in depression research. If our hypothesis holds true that the EMC strategy leads to a relevantly higher proportion of remitted patients, guidelines for the treatment of MDD could implement recommendations for adjusting an individual's treatment regularly during the early course of therapy, i.e., within the first 2 weeks of treatment.

Early and continued monitoring of treatment outcome is no standard application in clinical practice: Superiority of the EMC strategy could change clinical practice profoundly. Clinicians would be prompted to optimise their treatment in case of non-improvement after 2 weeks because of clear evidence that non-improvers with an early treatment optimisation would be more likely to become remitters compared to those patients with continued medication. Early and continued monitoring of treatment outcome would become a standard application in MDD treatment because it can be easily implemented in the clinical setting. It does not require expensive technical investment and can be applied worldwide. Implementation only requires an assessment of depression severity at baseline and at weekly intervals with an adequate scale. Given the potential for saving time and costs by using early improvement as a predictor of later outcome, a weekly investment of 15-20 minutes for the rating of depressive symptoms would be reasonable.

The economic effects of EMC could be substantial since patients could be cured from depression in a shorter period of time, which could decrease direct and indirect costs associated with MDD. Furthermore, this could substantially decrease the duration of prescriptions of antidepressants with limited effectiveness for the individual patient. The continued prescription of an inefficient drug can result in a substantially prolonged treatment duration, which highly increases resource use. However, the evaluation of the economic effects of the EMC strategy compared to TAU is not the main focus of the EMC trial. The selected instruments SF-12 as clinician and self report interview will allow the assessment of differences in quality of life between EMC and TAU. Furthermore, exploratory analyses like the calculation of differences in the duration of hospital treatment between EMC and TAU will give first suggestive information on the economic effects of EMC strategy. Studies for the comprehensive analysis of the economic effects of an EMC strategy in major depression should be conducted in case of a proven clinical superiority of EMC vs. TAU.

In research settings the typical duration of trials for the evaluation of ADs' efficacy is between 4-8 weeks, or even longer. E.g., the STAR*D trial has been conducted with a duration of a single medication for up to 14 weeks [[9], and references inside]. In MDD, more research trials for the evaluation of antidepressants' efficacy would use a shorter period than the 4-8 weeks currently regarded as appropriate. The EMC strategy would be extended to other antidepressants in the treatment of MDD. Additionally, the evaluation of the EMC strategy would likely be extended to other major psychiatric disorders like schizophrenia or generalised anxiety disorder. Regarding schizophrenia, it has been shown that a true drug response to antipsychotic pharmacotherapy can be observed within 2 weeks of treatment [40,41]. Regarding generalised anxiety disorder, retrospective analysis of time courses of response to drugs with largely different pharmacologic profiles (duloxetine, benzodiazepines, and serotonin receptor 1A partial agonist) showed that early improvement of anxiety symptoms was highly predictive of later outcome [42].

As described above, current guidelines recommend a treatment optimization after 4 weeks in case of insufficient outcome (<50% symptom reduction) after 4 weeks of treatment [15]. After this treatment optimisation step, there are no further recommendations on the appropriate monitoring or further measures for treatment optimisation. Therefore, the EMC trial will be able to give valuable information regarding this significant gap in the knowledge about depression treatment. Level 2 of the EMC trial addresses patients, who show an initial improvement, but do not experience a response after 4 weeks of treatment. For these patients, current guidelines recommend a treatment optimisation. These recommendations will be adhered to by a switch to venlafaxine treatment. After this, guidelines do no include recommendations on the monitoring or further treatment optimisation steps in the course of treatment. Therefore, we will apply an EMC strategy (EMC 2) and a standard strategy (TAU 2) to patients not improving after 2 weeks of venlafaxine treatment. Level 3 of the EMC trial addresses patients, who show an initial improvement, experience a response after 4 weeks of treatment, but get stuck in their symptom amelioration between weeks 4 and 6 of treatment. For these patients, current guidelines do not contain any treatment recommendation. Therefore, we will apply an EMC strategy (EMC 3) and a standard strategy (TAU 3) to these patients.

In summary, The EMC Trial will provide evidence regarding substantial questions in the treatment of major depression. Superiority of the EMC strategies compared to the respective TAU strategies could have substantial impact on patients' welfare, clinical guidelines, daily clinical practice, and economic costs of MD as well as research settings.

## Competing interests

The authors declare that they have no competing interests.

## Authors' contributions

KL and AT developed the idea of the trial. AT, SG, ND, CH, DFB, JR, DVC, KK, TG, MSG, KL participated in the conception and design of the trial. DW is the trial statistician and he made substantial contributions to the study design. AT and SG wrote the study protocol. AT drafted the manuscript. All authors critically reviewed and approved the final version of the manuscript. The corresponding author had final responsibility for the decision to submit for publication.
